# P10 Treating osteoporosis, facing reality – adopting a holistic approach

**DOI:** 10.1093/rap/rkab068.009

**Published:** 2021-10-19

**Authors:** Rifat Mazumder, Zakia Sultana

**Affiliations:** 1 East Suffolk and North Essex NHS Foundation Trust (Ipswich Hospital), Ipswich, United Kingdom; 2 Queen Elizabeth Hospital King's Lynn NHS Foundation Trust, Kings' Lynn, United Kingdom

## Abstract

**Case report - Introduction:**

Osteoporosis is a significant health problem; globally around 200 million women are affected. In Europe, osteoporosis is responsible for a higher disability encumber than cancer (with the exception of lung cancer). The treatment of osteoporosis is quite exacting. Although understanding of the diagnosis and treatment of osteoporosis has broadened considerably over the last few years, lack of bridging information still exists with guidance lacking on the appropriate management of complex comorbid clinical scenarios.

Here we will present a scenario of a patient with osteoporosis and multiple risk factors and comorbidities, where choice of suitable anti osteoporotic treatment was quite challenging.

**Case report - Case description:**

An 84-year-old lady with known osteoporosis with history of T-12 fracture (in 2009), sarcoidosis, coeliac disease (confirmed on duodenal biopsy), chronic hepatitis, history of acute kidney injury secondary to zoledronic acid infusion and urosepsis in 2017 was re-referred to the rheumatology clinic from respiratory team for optimisation of her bone protection.

She was previously on risedronate for almost 5 years without any improvement of bone mineral density. She was last seen in rheumatology in 2018, because of ineffectiveness and intolerance to alendronate (gastritis) and intravenous zoledronate – a discussion about subcutaneous denosumab was had, but the patient refused that option because of needle phobia. So, the plan was to maintain her on optimisation of vitamin D and calcium level. She was discharged from the clinic. Her GP advised her against vitamin D or calcium supplements because of episodes of hypercalcaemia secondary to sarcoidosis. For the last 3 years she was not on any bone protection or even calcium or vitamin D supplements.

Recently she noticed a worsening of exertional dyspnoea. She was reviewed by the respiratory team. Her lung function test showed slow progression of restrictive lung disease with FEV1/FVC ratio is 100.4%. In December 2020, she was started on prednisolone 30mg, which gradually stepped down; at the moment, she is on 15mg and will continue it as maintenance.

The patient was an ex-smoker, and drinks alcohol at about 10 unit/week. Her mobility is slightly better compared to the last few years. She trys to keep active and is enjoy gardening in the sunny weather.

It was difficult to convince her for blood tests; however, we succeeded after repeated counselling. Her blood tests showed microcytic anaemia, with normal inflammatory markers mild renal impairment with eGFR of 67, corrected calcium 2.19, alkaline phosphatase 78, vitamin D 49 (sub optimal) albumin 32.

**Case report - Discussion:**

Considering her age, comorbidities, frailty, intolerance and doubt about the efficacy, selecting an appropriate bone protection for her was fairly hard. Starting denosumab had more risk than benefit and in future if it need to stop there is an increased chance of rebound fracture. Besides this, she re-expressed her reluctance to the subcutaneous option. Moreover, calcium and vitamin D level were low in her recent blood tests. She did not fulfil the criteria for considering teriperatide.

We reviewed her DEXA scan in 2018, which showed an overall 19% reduction of BMD compared to 2009 (1.6% per year). She was on risedronate intermittently for about 4 years that time; however, she had not experienced any new fracture at that point. She had multiple hospital admissions during those years. Bone protection was withheld multiple times. Poor mobility, frailty status and other comorbidities during that period were also responsible for BMD decline.

Her case was discussed with a consultant with special interest in metabolic bone disease. Treatment decisions should be individualised; risk versus benefit needs to be considered to ensure the best outcome for the patient. We have decided to put her back on risedronate for at least 3 years. She tolerated only this medication in the past. We have requested bone markers and a repeat DEXA scan.

**Case report - Key learning points:**

Comorbidities adversely affect the management of osteoporosis. A comprehensive assessment of the comorbid list is necessary before considering changing a medication which suits the patient well and when there is limited option. Obstacles to offer high quality service are knowledge, expertise, and critical thinking from healthcare professionals, and knowledge and compliance to treatment from patients. Facing those challenges and treating patients judiciously will help to reduce the potential health and economic burden of osteoporosis.

